# An Efficient Gradient-Free Topology Optimization Method Based on Superellipse Curves and a Multilayer Perceptron Surrogate

**DOI:** 10.3390/mi17091074

**Published:** 2026-09-10

**Authors:** Fengyi Jin, Yanli Liu

**Affiliations:** College of Electrical and Control Engineering, Liaoning Technical University, Huludao 125105, China; 4724200301@stu.lntu.edu.cn

**Keywords:** gradient-free optimization, nonlinear materials, superellipse curve, surrogate model, topology optimization

## Abstract

Topology optimization is an effective method for obtaining high-performance material distributions with novel configurations. However, its application to complex electromagnetic devices remains challenging because of the difficulty of deriving sensitivities and the low computational efficiency associated with repeated finite element method (FEM) evaluations for nonlinear materials. This paper proposes an efficient gradient-free topology optimization method that integrates superellipse curves with a multilayer perceptron (MLP) surrogate model while accounting for nonlinearity. First, based on the general superellipse curve, an improved expression is introduced, in which the size, shape, and position can be flexibly controlled by only seven parameters. Then, a parameterized superellipse-curve-based gradient-free topology optimization framework is established, which can be applied to complex electromagnetic devices with nonlinear materials and complex objective functions. Moreover, a lightweight MLP-based surrogate model is constructed using limited training samples generated by Latin hypercube sampling and FEM, and it can replace FEM for evaluating nonlinear material behavior with negligible computational cost. Finally, the proposed topology optimization framework is applied to the design of a magnetic actuator, both with and without considering nonlinear B–H characteristics, demonstrating its effectiveness.

## 1. Introduction

Topology optimization has become an effective computational design tool for automatically generating high-performance material layouts within a prescribed design domain subject to specified objectives and constraints [1,2]. Unlike conventional size or shape optimization, which usually relies on predefined configurations and engineering experience, topology optimization can explore material distributions with much greater design freedom [3]. Therefore, it has been widely applied to structural, thermal, fluid, acoustic, and electromagnetic systems [4,5,6]. In electromagnetic devices, such as magnetic actuators and electric machines, the material layout of ferromagnetic components strongly affects magnetic flux paths, local saturation, leakage flux, force, and torque characteristics. Consequently, topology optimization has attracted increasing attention as a promising approach for improving electromagnetic performance and material utilization [7,8].

Classical topology optimization methods mainly include density-based methods and level-set methods. The solid isotropic material with penalization (SIMP) method describes the design domain using element-wise pseudo-density variables, which are updated based on sensitivity information [9]. The level-set method implicitly represents material boundaries and evolves them based on shape derivatives [10]. These methods have achieved significant success in continuum topology optimization. However, most conventional approaches rely on accurate sensitivities of the objective functions and constraint functions with respect to the design variables. For simple electromagnetic devices with differentiable objectives, such sensitivities can be efficiently obtained using the adjoint variable method [11]. Nevertheless, in electromagnetic devices, nonlinear magnetic materials and complex performance indices make the derivation of sensitivities difficult and highly problem-dependent.

This difficulty is particularly evident in nonlinear electromagnetic topology optimization. Ferromagnetic materials generally exhibit nonlinear B–H characteristics, and their permeability varies with the local magnetic flux density [12]. As a result, the finite element method (FEM) equations become nonlinear and usually require Newton–Raphson iterations or successive linearization to obtain a converged magnetic field solution [13,14]. Although these numerical schemes are effective for field analysis, they increase the computational cost of each simulation and further complicate the calculation of analytical sensitivities. In addition, the objective functions of electromagnetic devices may involve braking torque, magnetic force, flux-density uniformity, saturation suppression, or leakage-flux reduction, rather than being simple energy-based functions [15,16]. These objective functions are often nonlinear and difficult to differentiate with respect to a large number of topology variables. Therefore, conventional gradient-based topology optimization methods are not always suitable for nonlinear electromagnetic design problems.

Gradient-free optimization provides an alternative because it requires only objective-function evaluations and can be coupled with FEM solvers in a black-box manner [17,18]. Genetic algorithms, particle swarm optimization, differential evolution, and other heuristic methods have been widely used for nonlinear and multimodal engineering optimization problems [19,20]. However, directly applying these methods to topology optimization is usually inefficient because of the high dimensionality of the design space. For example, the normalized Gaussian network represents a topology using several Gaussian basis functions, whose positions, radii, and weights are optimized [21]. Such parameterized topology optimization methods avoid explicit sensitivity derivation and can naturally accommodate nonlinear materials and complex objective functions. Nevertheless, each basic Gaussian component is essentially circular and has limited boundary deformation capability. Therefore, many Gaussian functions may be required to describe complex geometries, which increases the number of design variables and reduces optimization efficiency. Another gradient-free topology optimization method based on projective transformations and Boolean operations (PTBOs) has also been proposed [22], in which basic components such as rectangles, triangles, and circles are transformed through scaling, rotation, shearing, translation, and projection. Note that the topology of each basic component remains unchanged during transformation. Such parameterized methods can simplify the topology optimization process, but the limited deformation capability and large number of control parameters of the basic components restrict further development. In our previous study [23], a superellipse-based gradient-free topology optimization method for magnetic actuator design was proposed, in which each material boundary was described by seven geometric parameters. However, the method relied on direct, time-consuming FEM evaluations and was limited to linear materials, restricting its further application.

During population-based optimization in gradient-free topology optimization, hundreds or even thousands of candidate topologies may need to be evaluated, which is computationally demanding. For nonlinear electromagnetic devices, each candidate requires an iterative FEM solution, with the magnetic permeability updated repeatedly until convergence is reached [24]. As a result, purely simulation-driven heuristic optimization is often computationally expensive and may be unsuitable for rapid engineering design. Surrogate modeling provides an effective way to alleviate this computational burden [25,26]. By learning the mapping from design variables to objective responses from a limited number of high-fidelity samples, a surrogate model can replace most of the expensive simulations during the optimization process. In particular, an ideal surrogate model should be lightweight and require only a small number of input samples.

In Ref. [27], a genetic algorithm (GA)-based topology optimization method was accelerated using a convolutional neural network (CNN) surrogate that maps binary topology images directly to the performance of an electromagnetic actuator. After training, the CNN provides rapid performance predictions and reduces reliance on repeated electromagnetic simulations. Similar image-based surrogate modeling studies can be found in [28,29]. However, these benefits come at the expense of substantial offline costs: generating a sufficiently representative image dataset requires numerous high-fidelity simulations, while the high-dimensional inputs make network training and tuning relatively complicated. The trained model may also need to be reconstructed when the design resolution or operating conditions change. In addition, a transformation from the input binary topology images to CAD-ready topologies is required, limiting the transferability of the method and increasing the overall implementation burden.

Moreover, Lei Xu combined the moving morphable component (MMC) method with a deep neural network (DNN) and transfer learning to efficiently predict acoustic responses and facilitate the topology optimization of serialized acoustic structures [30]. However, constructing the predictive model still relies on a substantial number of high-fidelity training samples. When the design conditions change considerably, additional data collection and model adaptation or reconstruction may be required. Wesley C. Dossett [31] integrated a residual neural network, a CNN, and reinforcement learning to achieve efficient sound-power topology optimization of shell structures. This framework provides strong capabilities for extracting spatial features and exploring the design space. Nevertheless, its multistage learning architecture increases the complexity of model training and hyperparameter tuning. Moreover, prediction errors introduced by the surrogate model may propagate through the subsequent learning and optimization stages, while the generalization capability of the resulting model remains dependent on the coverage of the training-data distribution.

To address these challenges, this study proposes an efficient gradient-free topology optimization method that integrates improved superellipse curves with a multilayer perceptron (MLP)-based surrogate model while accounting for material nonlinearity. In the proposed method, material boundaries are represented by improved superellipse curves, each controlled by only seven parameters. These parameters are directly employed as the inputs to a lightweight MLP surrogate model. Consequently, the proposed method avoids the high-dimensional inputs associated with topology images, as well as the additional procedures required for image generation and feature mapping. The MLP directly approximates the mapping from the geometric design variables to the electromagnetic performance, and its input parameters correspond explicitly to the CAD geometry of the topology. This correspondence also facilitates the reconstruction and subsequent engineering implementation of the optimized geometry. Using a limited number of training samples generated through Latin hypercube sampling and evaluated using FEM, the surrogate substantially reduces the number of FEM evaluations required during the subsequent gradient-free optimization. Furthermore, because no analytical sensitivity derivation is required, nonlinear electromagnetic materials characterized by nonlinear B–H characteristics, together with complex objective functions, can be incorporated directly into the optimization framework. Therefore, the advantages of the proposed method extend beyond reducing the online cost of design evaluation: its compact geometric parameterization and lightweight surrogate architecture also reduce the overall burden associated with data preparation, model training, and engineering implementation. Finally, the trained surrogate model is coupled with a genetic algorithm to rapidly predict the objective values of candidate topologies and efficiently search for the optimal material distribution.

## 2. Magnetic Actuator Model

**Figure 1:**
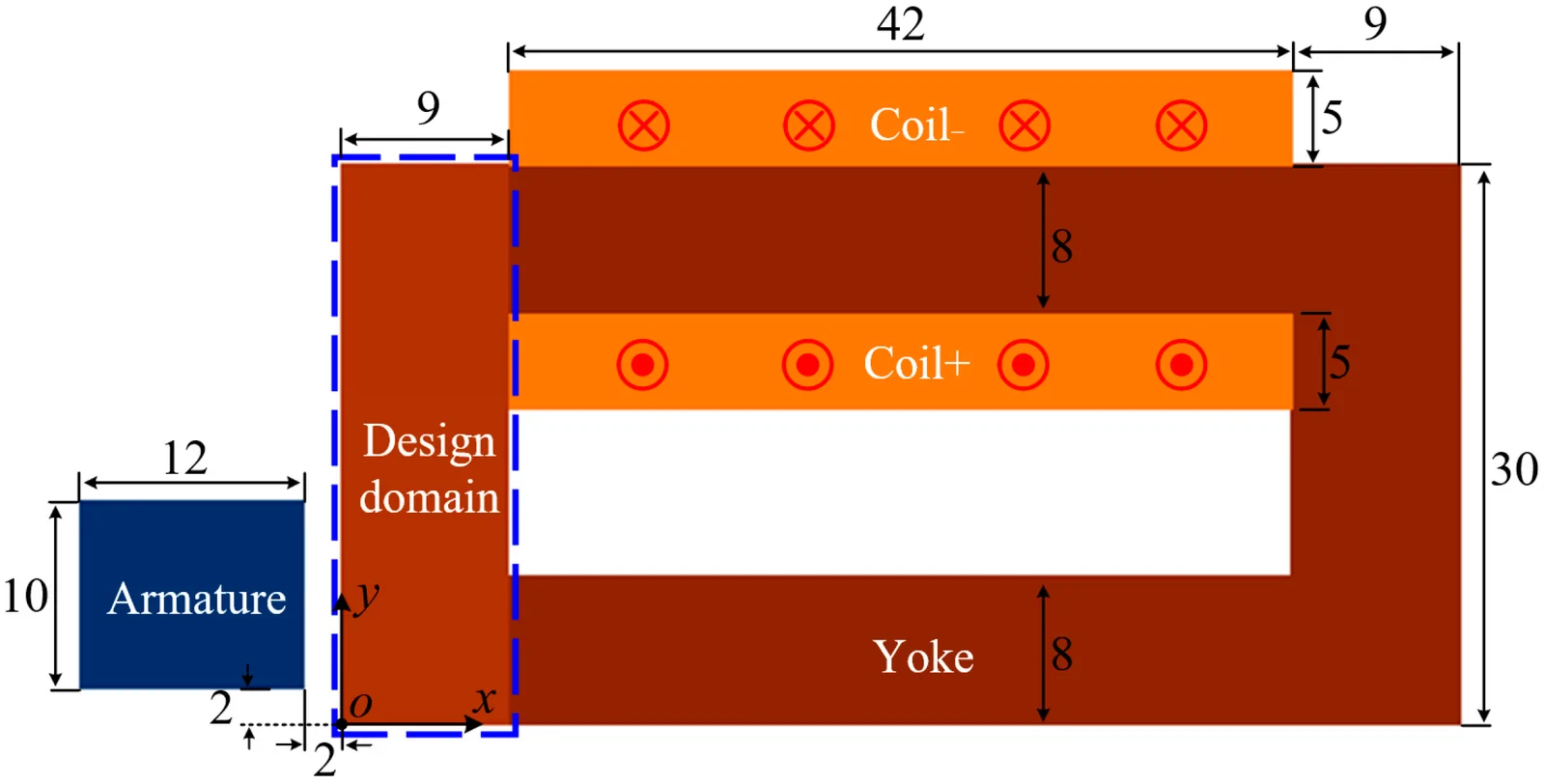
Schematic of the magnetic actuator. The yoke topology within the design domain is to be optimized to maximize the magnetic force of the armature (unit: mm).

The magnetic actuator model under investigation is shown in Figure 1. To attract the armature in the *x* direction, the coil is wound around the yoke to generate a magnetic field in the armature region and thereby produce a magnetic force along the *x* direction. The coil has a current density of 2 A/mm^2^ in the *z* direction. Because the yoke within the design domain plays an important role in guiding the magnetic flux toward the armature region, its topology should be carefully designed to maximize the magnetic force.

**Figure 2:**
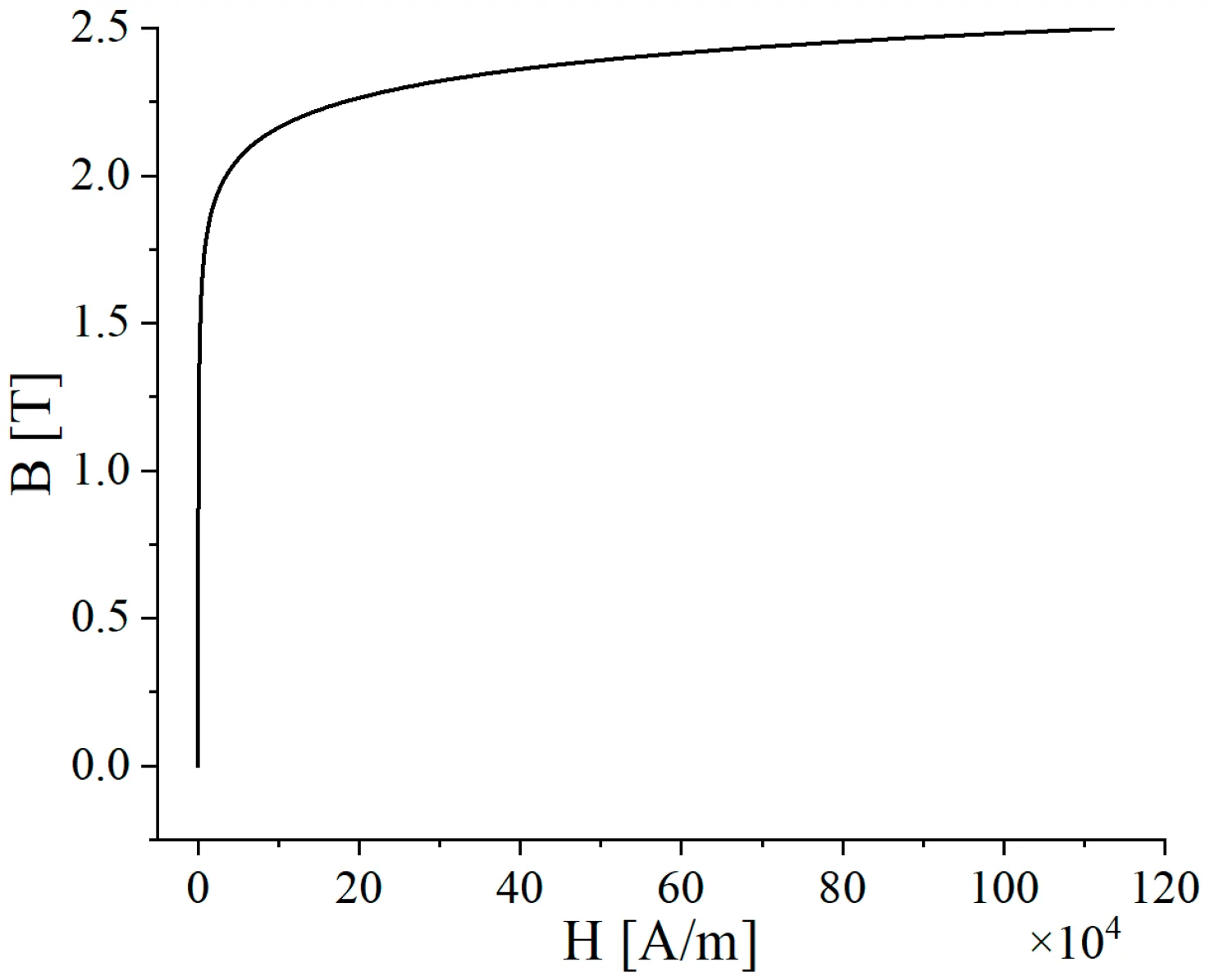
The nonlinear B–H curve of the yoke.

In particular, magnetic saturation is considered; that is, the nonlinear magnetic permeability of the yoke must be accounted for during the magnetic field calculation. Here, the approximated nonlinear permeability relation from [8] is adopted and expressed as follows:

(1) $$\mu (B^{2})=1/(q_{1}e^{{q_{2}}^{B^{2}}}+q_{3})$$

where *q*_1_, *q*_2_, and *q*_3_ are the parameters determining the characteristics of the B–H curve. Here, *q*_1_ = 49.4, *q*_2_ = 1.46, and *q*_3_ = 520.6 are adopted, and the B–H curve is presented in Figure 2.

Since the magnetic actuator can be simplified to a two-dimensional (2D) model and variation along the *z*-direction can be neglected, 2D finite element analysis is employed to solve for the magnetic field. By adopting the magnetic vector potential ***A*** as the primary variable, the governing equation is expressed as follows:

(2) $$\nabla \times (\frac{1}{\mu }\nabla \times A)=J$$

where *μ* denotes the magnetic permeability, and ***J*** represents the current density in the coil. In this 2D formulation, only the *z*-components, *J_z_* and *A_z_*, are considered. Using the Galerkin method, the finite element equation is obtained as follows:

(3) $$KA_{z}=B$$

where **K** denotes the stiffness matrix, and **B** is the right-hand-side vector. These are computed as follows:

(4) $$\left\{\begin{array}{l} K=\iint_{s}\frac{1}{\mu }(\frac{\partial A_{z}}{\partial x}\frac{\partial N}{\partial x}+\frac{\partial A_{z}}{\partial y}\frac{\partial N}{\partial y})dxdy\\ B=\iint_{s}J_{z}Ndxdy \end{array}\right.$$

where *N* is the shape function of the finite element. After solving for the magnetic vector potential ***A***, the magnetic field ***B*** can then be obtained:

(5) B=∇×A

In particular, the permeability of the yoke varies as a function of the magnetic flux d the magnetic flux density ***B***. To account for this magnetic saturation characteristic in the finite element model, the successive linearization method is adopted. Accordingly, at each iteration, the permeability of each yoke element is updated according to the following equation:

(6) $$\mu^{\mathrm{new}}=\mu^{\mathrm{old}}+\omega (\mu^{{\mathrm{new}}^{\prime }}-\mu^{\mathrm{old}})$$

where $\mu^{\mathrm{old}}$ represents the permeability from the previous iteration, $\mu^{{\mathrm{new}}^{\prime }}$ denotes the permeability obtained by combining the solution from the previous iteration with the nonlinear B–H curve, and ω is the relaxation factor. In this work, ω is set to 0.5 to ensure both convergence and computational efficiency. In addition, the initial relative permeability of all yoke elements is set to 1000, and the iteration process is terminated when the average relative change in permeability between two consecutive iterations falls below a prescribed threshold.

It should be noted that accounting for the nonlinear B–H characteristic substantially increases the computational time. Typically, approximately 10 iterations are required to obtain a relatively stable solution for a single magnetic actuator model.

After obtaining the magnetic field solution, the magnetic force acting on the armature is calculated using the Maxwell stress tensor method for accuracy and efficiency. The magnetic force is calculated as follows:

(7) $$F=\int_{S}T\cdot nds$$

where ***T*** is the Maxwell stress tensor, and ***n*** is the outward unit normal vector on the integration surface *S*. For the 2D magnetic actuator model, the above surface integral reduces to a line integral. In this study, only the magnetic force component along the *x* direction, *F_x_*, is considered.

## 3. Gradient-Free Topology Optimization Model

For the magnetic actuator, the nonlinear B–H characteristic makes it difficult to apply conventional topology optimization methods, such as density-based and level-set methods, because the required sensitivities are challenging to derive analytically.

In essence, the primary objective of magnetic actuator optimization is to determine the optimal yoke topology within the design domain. Therefore, if a wide range of yoke topologies can be represented using only a few parameters, gradient-free topology optimization can be performed efficiently to identify the optimal yoke topology.

In practice, various geometric primitives can be used to represent the yoke topology, such as rectangles, circles, and triangles. However, both the boundary deformation capability and the number of control parameters must be considered. In this study, superellipse curves are adopted to represent material boundaries. As a generalization of the ellipse, a superellipse curve can represent a wide range of basic boundary forms, including circles, squircles, and rectangles, through simple adjustments of its control parameters. In the Cartesian coordinate system, the algebraic formulation of the superellipse curve is given as follows:

(8) $${\left|\frac{x}{a}\right|}^{\frac{2}{p}}+{\left|\frac{y}{b}\right|}^{\frac{2}{q}}=1$$

where *x* and *y* are the coordinates of a point on the superellipse curve, and *a* and *b* are the semi-axes that control its size. The parameters *p* and *q* denote the squareness of the curve and determine its boundary form. Notably, the superellipse curve is defined by only four control parameters. Based on Equation (8), the coordinates of points on the superellipse curve can be expressed as follows:

(9) $$\left\{\begin{array}{l} x(\theta )={\left|\cos(\theta )\right|}^{p}\cdot a\cdot \mathrm{sgn}(\cos(\theta ))\\ y(\theta )={\left|\sin(\theta )\right|}^{q}\cdot b\cdot \mathrm{sgn}(\sin(\theta )) \end{array}\right.,\;\theta \in [0,\;2\pi ]$$

where sng() denotes the sign function. Therefore, the boundary of the superellipse curve can be explicitly described and subsequently used to represent the material boundaries in the topology optimization process.

However, the boundary deformation capability of the original superellipse curve still needs to be improved. First, a rotation operation is introduced into the original superellipse curve. Let *α* denote the rotation angle. For a point with the original coordinates (*x*, *y*) on the initial superellipse curve, the coordinates of the rotated point can be obtained through the following matrix transformation:

(10) $$\left[\begin{array}{c} x_{1}(\theta )\\ y_{1}(\theta ) \end{array}\right]=\left[\begin{array}{cc} \cos(\alpha ) & -\sin(\alpha )\\ \sin(\alpha ) & \cos(\alpha ) \end{array}\right]\times \left[\begin{array}{c} {\left|\cos(\theta )\right|}^{p}\cdot a\mathrm{sgn}(\cos(\theta ))\\ {\left|\sin(\theta )\right|}^{q}\cdot b\mathrm{sgn}(\sin(\theta )) \end{array}\right],\;\theta \in [0,\;2\pi ]$$

Notably, introducing the rotation operation requires only one additional parameter, yet it significantly enriches the range of representable topologies.

Up to this point, the center of the superellipse curve has been fixed at the origin (0, 0). In practice, allowing the center of the superellipse curve to vary can further enrich the range of representable topologies. To this end, two additional parameters, (*x*_0_, *y*_0_), are introduced to control the centre of the superellipse curve. As a result, the coordinates of a point on the fully specified superellipse curve can be expressed as:

(11) $$\left[\begin{array}{c} x_{2}(\theta )\\ y_{2}(\theta ) \end{array}\right]=\left[\begin{array}{cc} \cos(\alpha ) & -\sin(\alpha )\\ \sin(\alpha ) & \cos(\alpha ) \end{array}\right]\times \left[\begin{array}{c} {\left|\cos(\theta )\right|}^{p}\cdot a\mathrm{sgn}(\cos(\theta ))\\ {\left|\sin(\theta )\right|}^{q}\cdot b\mathrm{sgn}(\sin(\theta )) \end{array}\right]+\left[\begin{array}{c} x_{0}\\ y_{0} \end{array}\right],\;\theta \in [0,\;2\pi ]$$

Based on the above analysis, the improved superellipse curve is fully characterized by only seven parameters: *a*, *b*, *p*, *q*, *α*, *x*_0_ and *y*_0_. In other words, when this superellipse curve is adopted to represent the material topology, diverse topologies can be generated simply by adjusting these seven control parameters.

To further enhance the capability of the superellipse curve to represent more complex topologies, Boolean operations are introduced. Specifically, the union of multiple superellipse curves can be used to represent much more complex material topologies. Since each superellipse curve is controlled by only seven parameters, the total number of design parameters remains relatively small even when the Boolean union of multiple superellipse curves is considered.

**Figure 3:**
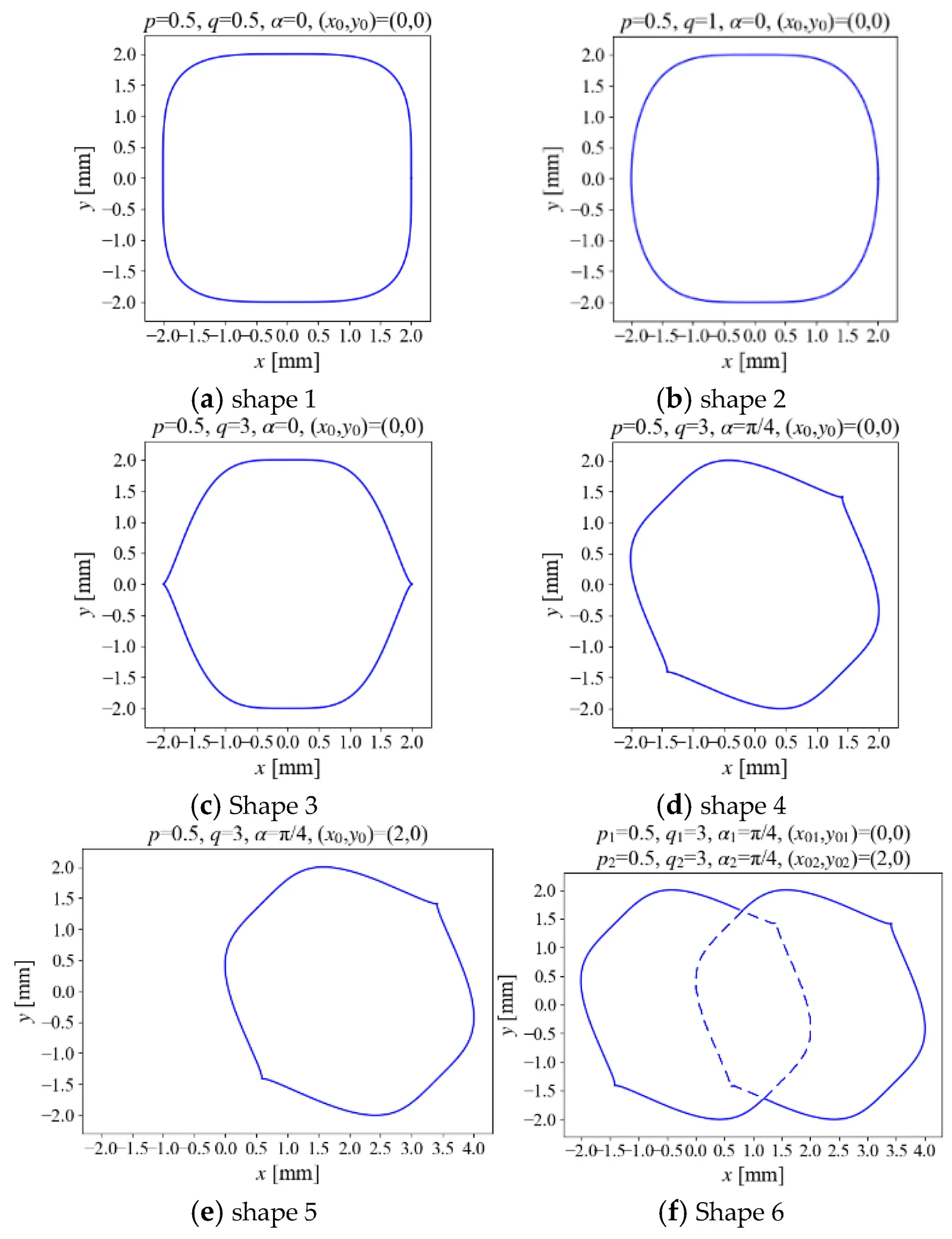
Superellipse curves under different control parameters, and the solid blue lines represent the boundaries of the topologies.

To demonstrate the deformation capability of the proposed superellipse curves, Figure 3 presents the topologies generated under different control parameters, with the semi-axis lengths fixed at *a* = *b* = 2 in all cases. In Figure 3a, when *p* = *q* = 0.5, an approximately rectangular topology is obtained, although the corners remain relatively rounded. In Figure 3b, the corners become more rounded as *q* increases from 0.5 to 1. Furthermore, the directional difference becomes more pronounced in Figure 3c, where q is further increased to 3.

Figure 3d shows the deformed superellipse curve obtained by applying a rotation operation. Specifically, the curve is rotated counterclockwise by π/4, yielding a different topology. In Figure 3e, a translation operation is applied, shifting the centre of the superellipse curve from (0, 0) to (2, 0). Finally, Figure 3f presents the Boolean union of the two superellipse curves shown in Figure 3d,e. The resulting topology is indicated by the solid lines. It can be seen that a much more complex topology is obtained, which cannot be represented by a single superellipse curve.

Since the material topology is represented by the proposed superellipse curves, each described by only a limited number of parameters, these parameters can be efficiently optimized using heuristic methods such as genetic algorithms and particle swarm optimization. This parameterized representation offers an additional advantage in that it avoids the need to derive sensitivities of the objective function with respect to the design variables. For example, when nonlinear B–H characteristics, complex objective functions, or even multi-objective formulations are considered, direct sensitivity derivation is often difficult. In contrast, the proposed superellipse-curve-based gradient-free topology optimization method requires only the optimization of a small number of control parameters.

Additionally, the proposed topology optimization method is naturally compatible with the FEM. First, the entire magnetic actuator, including the design domain, is discretized only once. For each element in the design domain, if its centroid lies inside the superellipse-defined region, the element is assigned to material 1; otherwise, it is assigned to material 2. In this way, the material distribution, or equivalently the material topology, can be readily determined for each set of design parameters without repeated remeshing. Once the topology is defined, its performance can be evaluated using FEM.

Here, the objective function for the magnetic actuator in the topology optimization problem is defined as follows:

(12) $$E=W_{1}\frac{F_{x\_cal}}{F_{x\_ref}}+W_{2}\frac{e^{\beta (S_{ref}-S_{cal})}}{1+e^{\beta (S_{ref}-S_{cal})}}$$

where *F_x_cal_* represents the calculated magnetic force, *S_cal_* denotes the yoke area, *F_x_ref_* is the reference magnetic force, and *S_ref_* is the reference yoke area, which also serves as the upper bound for the yoke area. *S_ref_* is set to 60% of the total area of the design domain (corresponding to 162 mm^2^). Moreover, *W*_1_ and *W*_2_ are the weighting coefficients in the optimization objective, and both are set to 1 in our model. The second term in Equation (12) introduces a sigmoid function to penalize deviations in the yoke area, and the parameter *β* acts as a scaling factor that controls the sharpness of the sigmoid transition, and it is set to 1 in our model. Accordingly, the proposed superellipse-curve-based topology optimization problem can be formulated as follows:

(13) $$\begin{array}{ll} \mathrm{Find}\text{:} & \left[a_{1},b_{1},p_{1},q_{1},\alpha_{1},x_{01,}y_{01},\ldots ,a_{N_{s}},b_{N_{s}},p_{N_{s}},q_{N_{s}},\alpha_{N_{s}},x_{0N_{s},}y_{0N_{s}}\right]\\ \mathrm{Maximize}\text{:} & E=W_{1}\frac{F_{x\_cal}}{F_{x\_ref}}+W_{2}\frac{e^{\beta (S_{ref}-S_{cal})}}{1+e^{\beta (S_{ref}-S_{cal})}}\\ S.t.\text{:} & a_{i\_\min}\leq a_{i}\leq a_{i\_\max}\\ & b_{i\_\min}\leq b_{i}\leq b_{i\_\max}\\ & p_{i\_\min}\leq p_{i}\leq p_{i\_\max}\\ & q_{i\_\min}\leq q_{i}\leq q_{i\_\max}\\ & x_{0i\_\min}\leq x_{0i}\leq x_{0i\_\max}\\ & y_{0i\_\min}\leq y_{0i}\leq y_{0i\_\max},\;(i=1,2,\ldots ,N_{s}) \end{array}$$

where *N*_s_ denotes the number of superellipse curves used in the topology optimization framework.

Typically, for a given magnetic actuator topology, estimating the magnetic force using the FEM requires approximately 16.43 s on a standard laptop with a Python-based implementation (Python 3.10). When three superellipse curves are adopted, a total of 21 parameters must be optimized. If each parameter is assigned only 10 candidate values, the total number of possible parameter combinations reaches 10^21^, which is computationally prohibitive. In practice, the number of candidate values for each parameter is much larger than 10. Although heuristic optimization algorithms, such as genetic algorithms and particle swarm optimization, can identify relatively high-quality solutions while reducing the number of evaluated samples, the overall optimization process remains time-consuming.

## 4. Surrogate Model

Considering the prohibitive computational cost of directly performing magnetic actuator topology optimization using the FEM, a surrogate model is introduced to replace the time-consuming FEM and accelerate the optimization process. In this study, a multilayer perceptron (MLP) network is adopted and trained as the surrogate model.

**Figure 4:**
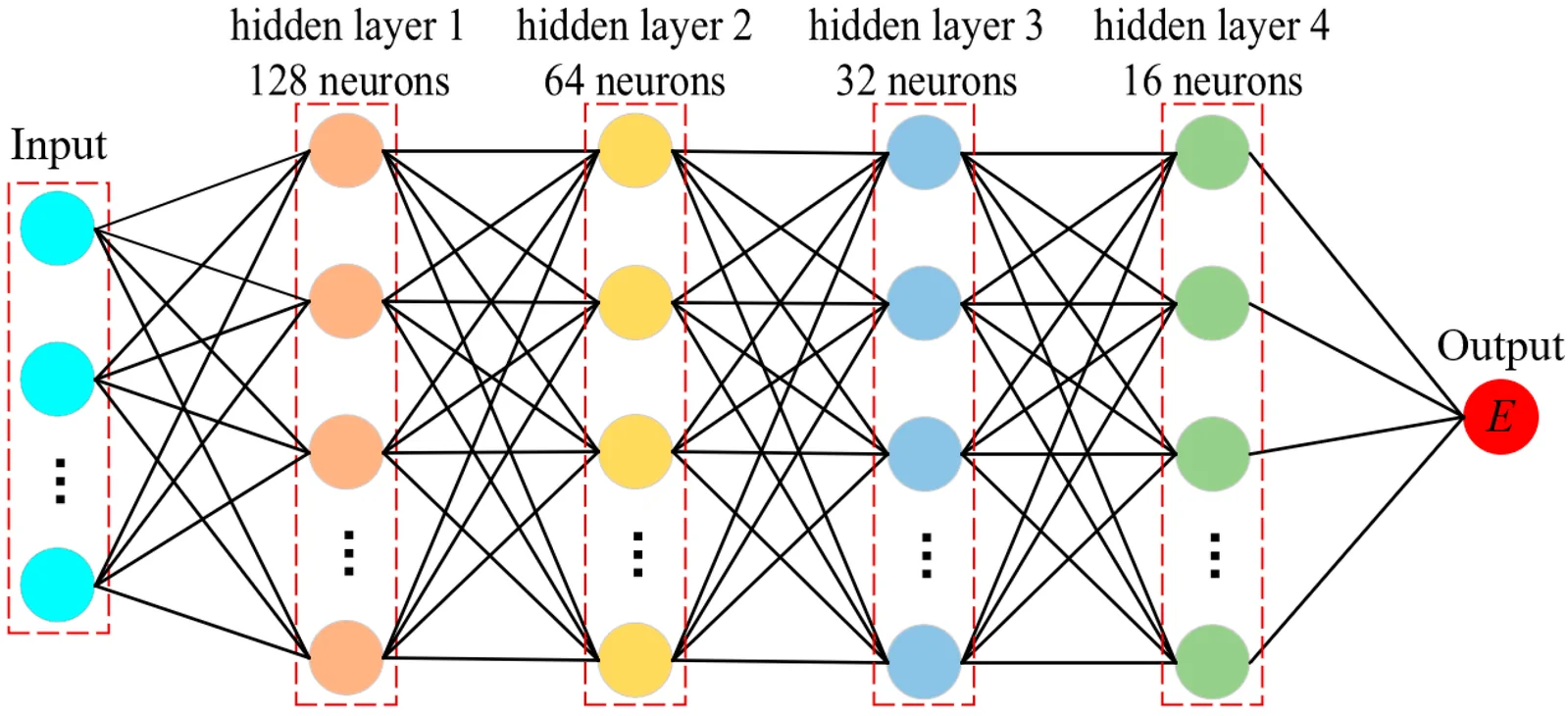
The schematic diagram of the MLP model architecture.

The MLP is a typical feedforward neural network composed of multiple neuron layers, including an input layer, one or more hidden layers, and an output layer. The schematic architecture of the MLP model used in this study is shown in Figure 4. Neurons in adjacent layers are connected by weights and biases learned during training, and activation functions are introduced to capture the nonlinear characteristics of the data. Compared with a traditional single-layer perceptron, the inclusion of hidden layers significantly improves the model’s ability to handle nonlinear multi-parameter prediction tasks.

In this work, the rectified linear unit (ReLU) is adopted as the activation function in the MLP framework. ReLU is a widely used activation function and is defined as *f*(*x*) = max (0, *x*). When the input is positive, the output remains linear; when the input is negative, the output is zero. Therefore, ReLU has the advantages of simple computation and strong nonlinear representation capability. Compared with traditional sigmoid or tanh activation functions, ReLU can effectively alleviate the vanishing-gradient problem, accelerate network convergence, and provide good training stability and generalization ability in high-dimensional nonlinear mapping tasks.

Furthermore, the Adam optimization algorithm, which is based on gradient descent, is used to update the parameters of the MLP model. Adam is an adaptive learning-rate optimization method that combines first-order and second-order moment estimates. It automatically adjusts the update step size for each parameter according to historical gradient information. Therefore, compared with conventional stochastic gradient descent, Adam generally provides faster convergence and better training stability.

Thus, in the MLP model, the inputs are the control parameters of the superellipse curves, and the output is the scalar weighted objective function defined in Equation (12). The samples were divided into training, validation, and test sets at a ratio of 80:10:10. The final MLP model consists of four hidden layers with 128, 64, 32, and 16 neurons, respectively, and uses SiLU activation functions. It was trained with the Adam optimizer using an initial learning rate of 5 × 10^−4^, a batch size of 512, and a maximum of 5000 epochs. The mean squared error was adopted as the loss function. The learning rate was reduced by a factor of 0.5 when the validation loss failed to improve for 800 epochs, and early stopping was applied according to the specified stopping criterion. Moreover, the number of input neurons depends on the number of superellipse curves; for example, when three superellipse curves are used, the total number of control parameters is 21, which is also the number of input neurons.

Obviously, providing as many samples as possible can improve the accuracy of the MLP model. However, generating a large number of training samples using FEM at the preliminary stage is computationally expensive and time-consuming. Therefore, in this work, Latin hypercube sampling (LHS) is employed to generate the input samples for MLP training. As a stratified sampling technique, LHS divides the range of each design variable into intervals with equal probability and ensures that samples are drawn from all intervals, thereby providing more uniform coverage of the multidimensional design space.

**Figure 5:**
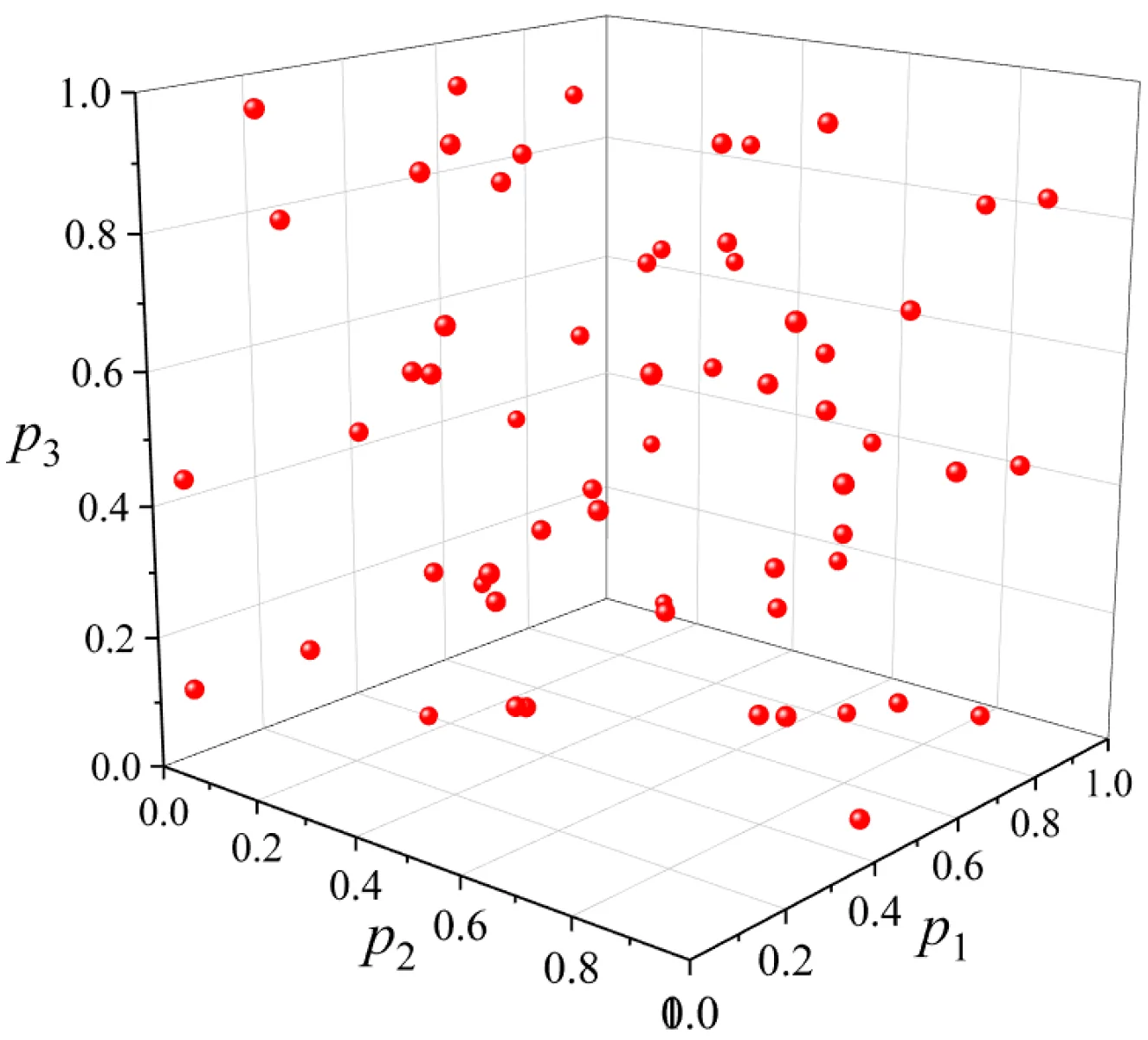
Distribution of 60 sampling points for three parameters based on LHS.

To further illustrate the advantage of the LHS method, Figure 5 presents the distribution of 60 sampling points for three parameters, *p*_1_, *p*_2_, and *p*_3_, each ranging from 0 to 1. It can be observed that the sampled points are uniformly distributed within the design domain. Therefore, the analysis of the entire design domain can be approximated by the analysis of a limited number of representative sampling points, which greatly simplifies the overall analysis.

For the magnetic actuator model considered in this study, the ranges of the control parameters for each superellipse curve during topology optimization are determined based on the dimensions of the design domain. On the one hand, the maximum size of each superellipse curve should be comparable to that of the design domain. On the other hand, the variation range of the superellipse curve should not excessively exceed the design domain, so as to avoid wasting the search space. Specifically, the allowable movement range of the center point is set to three times the size of the design domain. Moreover, to prevent excessive distortion of the superellipse curve, the ranges of both p and q are empirically selected as [0.1, 4]. Consequently, the ranges of all superellipse-curve parameters are summarized in Table 1.

The sampled points, or parameter combinations, correspond to a set of yoke topologies. The magnetic forces and yoke areas of these topologies are calculated using the FEM. These parameter combinations and their corresponding objective functions are then used to train the MLP model. Once the MLP model has been established, the objective function of a new parameter combination, or equivalently, a new yoke topology, can be predicted by the MLP model instead of being calculated using the FEM. In this way, the analysis time can be reduced from approximately 16.43 s to a negligible computational cost, providing a foundation for the efficient topology optimization of the yoke. The accuracy of the established MLP framework will be discussed in the Section 5.

It should be emphasized that, because the performance of the magnetic actuator is evaluated mainly at the numerical level based on FEM, the established MLP model is used to assess the fidelity of the surrogate with respect to the reference numerical model; it does not, by itself, validate the physical performance of the optimized magnetic actuator.

## 5. Optimization Results

In this section, the proposed topology optimization framework is applied to the design of the yoke in a magnetic actuator. For comparison, optimization results for both a linear yoke and a nonlinear yoke are presented.

### 5.1. Optimization Results Based on Linear Yoke

First, for comparison, a constant relative permeability of 1000 is adopted for the yoke. In this case, the computational time required for FEM is negligible compared with that required when nonlinear B–H characteristics are considered. Therefore, the FEM can be directly used to solve the magnetic field during optimization. The whole yoke design domain is discretized into 1864 triangular elements.

The genetic algorithm with an elite retention strategy is adopted for topology optimization. In this work, the area constraint for yoke optimization is set to 0.6. First, only one superellipse curve is used. The design domain is assumed to be initially filled with yoke material, while the region enclosed by the superellipse curve within the design domain is assigned as air. Therefore, different superellipse curves correspond to different yoke topologies in the design domain. The genetic algorithm is configured with a population of 350 individuals per generation and is run for 100 generations.

**Figure 6:**
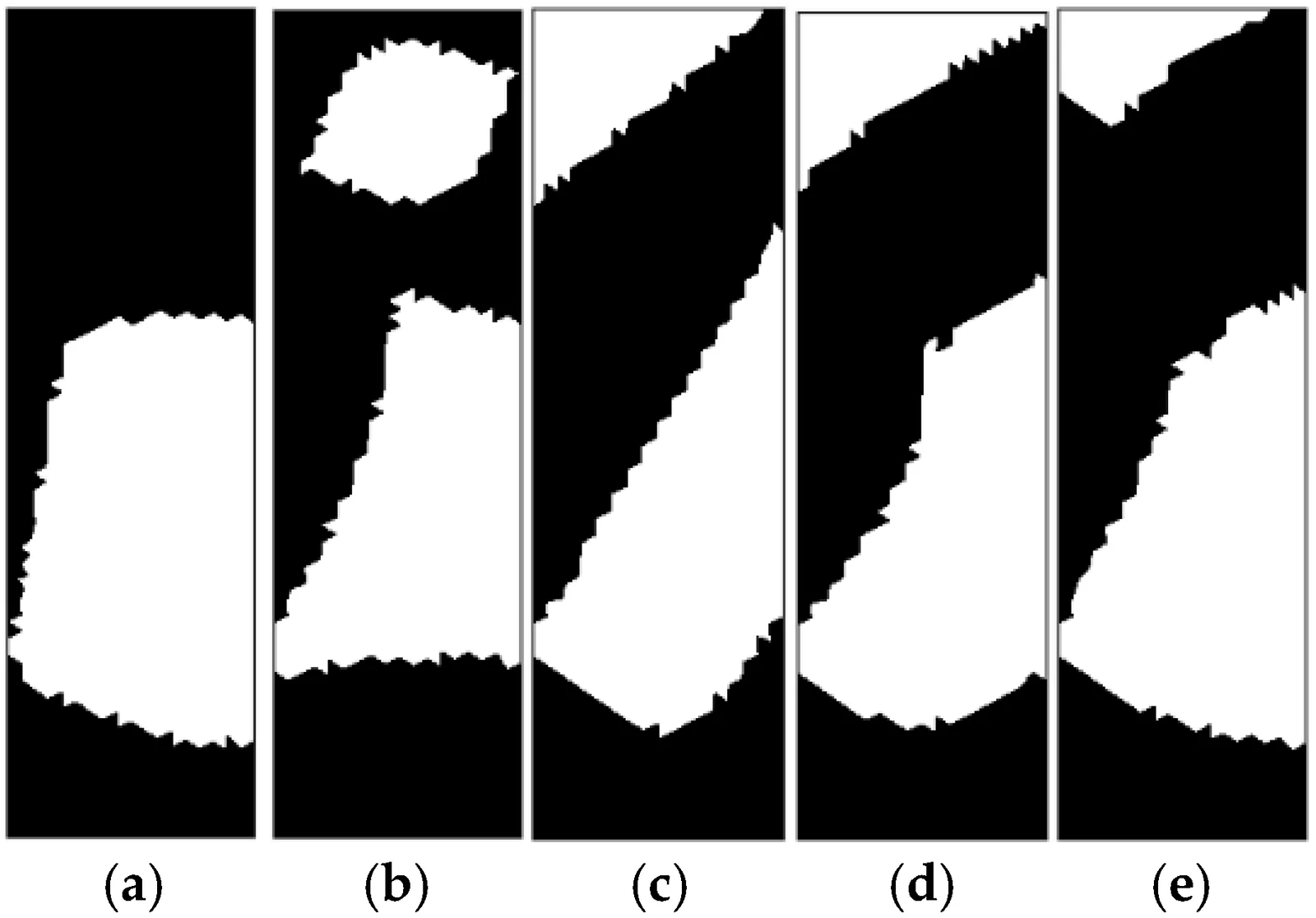
Optimized topologies of the yoke in a magnetic actuator under different cases, with the area constraint set to 0.6. (**a**) One superellipse curve. (**b**) Two superellipse curves. (**c**) Three superellipse curves. (**d**) Four superellipse curves. (**e**) Six superellipse curves.

Figure 6 presents the topology optimization results for the yoke in the magnetic actuator. Specifically, Figure 6a shows the result obtained using a single superellipse curve. The optimized yoke exhibits a C-shaped geometry. This topology is physically reasonable from an electromagnetic perspective: the magnetic flux generated by the upper coils is guided to the armature through the upper part of the yoke, while the lower part of the yoke within the design domain completes the magnetic circuit, thereby enhancing the magnetic force acting on the armature. The corresponding optimized magnetic force, *F_x_*, is approximately 52.32 N/m.

In addition, a larger number of superellipse curves is also considered in the topology optimization framework. For example, Figure 6b shows the topology optimization result obtained using two superellipse curves. The overall topology still resembles a C-shaped geometry, but with a hole at the center. The corresponding optimized magnetic force, *F_x_*, is approximately 52.58 N/m, which is slightly higher than the 52.32 N/m achieved with a single superellipse curve. Furthermore, the topology optimization results obtained using three, four, and six superellipse curves are presented in Figure 6c–e, respectively.

**Figure 7:**
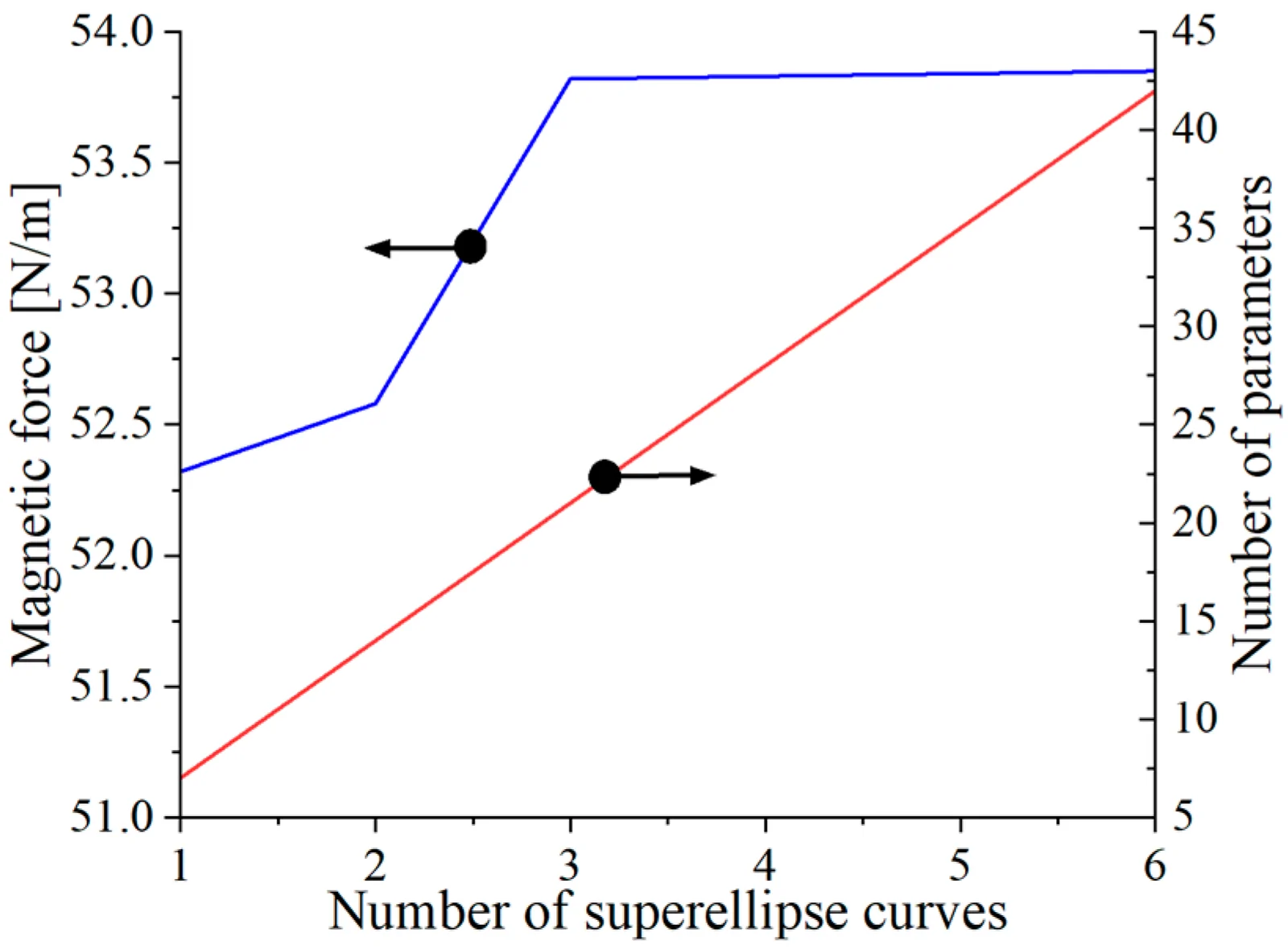
Optimized magnetic forces and the number of parameters change with respect to the number of superellipse curves. The blue line represents the magnetic force, and the red line indicates the number of parameters.

Based on the topology optimization results shown in Figure 6, the optimized designs exhibit similar overall topologies, all of which are generally C-shaped, although some local geometric details differ. Figure 7 illustrates the variations in the optimized magnetic force and the number of design parameters with respect to the number of superellipse curves used in the topology optimization. As the number of superellipse curves increases, the number of design parameters also increases. As indicated by the blue curve in Figure 7, the optimized magnetic force initially increases with the number of superellipse curves and then gradually levels off when approximately four curves are used, corresponding to 28 design parameters. The maximum magnetic force is about 53.83 N/m. Notably, further increasing the number of superellipse curves does not lead to a noticeable improvement in magnetic force, but it does increase the number of parameters and thus reduces optimization efficiency.

When performing superellipse-curve-based topology optimization in practice, one can start with an empirical number of superellipse curves, for example, two, and then gradually increase the number of curves, for example, by two each time, until the relative difference in the optimized material performance between two consecutive cases falls below a prescribed threshold. In this way, the optimal topology can be obtained more efficiently.

### 5.2. Optimization Results Based on Nonlinear Yoke

Here, the nonlinear B–H characteristic is taken into account in the yoke optimization. Based on the topology-optimization results for the linear-material case, four superellipse curves are sufficient to construct the topology optimization framework, striking a good balance between accuracy and computational efficiency. In this case, the total number of design parameters is 28, and 6000 uniformly distributed samples are generated using the Latin hypercube sampling (LHS) method, and the corresponding magnetic forces are evaluated using FEM.

**Figure 8:**
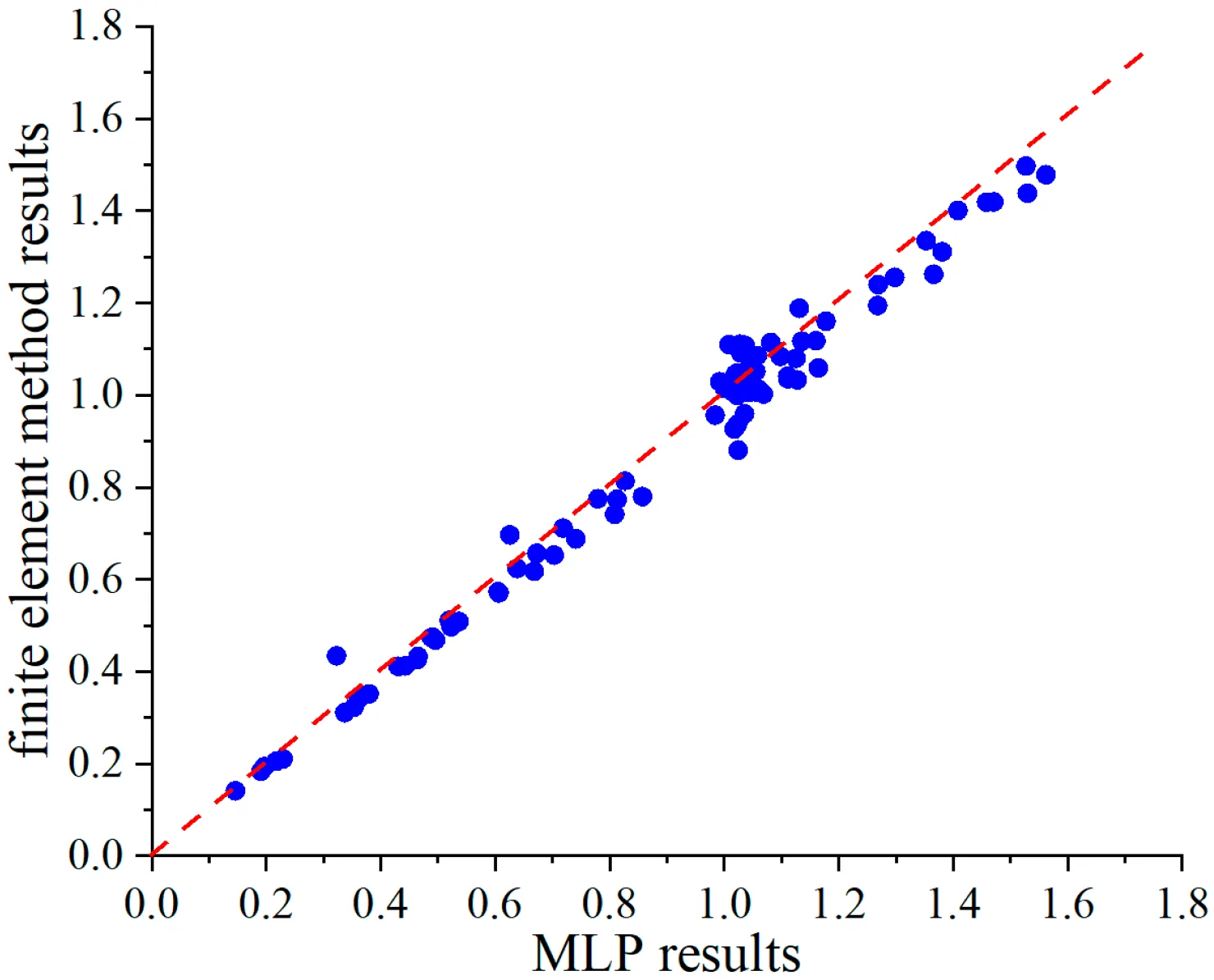
MLP model prediction results.

To evaluate the accuracy of the MLP model, some randomly distributed samples are generated using the Monte Carlo method (different from the training samples). Their magnetic forces are then predicted using both the MLP model and the FEM, and the comparison results are presented in Figure 8. Overall, the predicted results agree well with the reference results obtained by FEM. In particular, the Pearson correlation coefficient and the coefficient of determination of the established MLP model are 0.99 and 0.97, respectively.

To demonstrate whether 6000 training samples are sufficient, insufficient, or excessive, we considered 1000, 3000, 5000, 6000, and 9000 FEM samples, respectively, while keeping the network architecture, training settings, design-variable ranges, and random seed unchanged. An independent Monte Carlo dataset, not used for training or hyperparameter tuning, was employed to evaluate surrogate accuracy. The corresponding Pearson correlation coefficients were 0.945, 0.971, 0.987, 0.990, and 0.996, respectively. These results indicate that surrogate accuracy improves significantly as the number of training samples increases from 1000 to 9000. However, beyond 6000 samples, the improvement becomes marginal and does not lead to a meaningful enhancement in the FEM-verified optimization results. Therefore, 6000 samples were selected as a reasonable compromise between surrogate accuracy and the computational cost of generating the FEM training data.

As for the computational burden, for the conventional FEM, when the successive linearization method is used to solve the nonlinear magnetic actuator model on a standard laptop with a Python-based implementation, the time required for a single magnetic-actuator evaluation is approximately 16.43 s. With a genetic algorithm population of 350 individuals and 100 generations, the total optimization time is about 575,050 s. In contrast, preparing 6000 FEM samples and training the MLP model takes about 98,580 s and 204.30 s, respectively. Moreover, because the MLP model can predict all individuals in each generation simultaneously, the GA search time is only about 1 s. Therefore, the total time for the proposed MLP-assisted topology optimization method is approximately 98,785 s. Compared with the conventional FEM-based topology optimization time of 575,050 s, the proposed method saves about 82.8% of the total optimization time.

Although data preparation and MLP training require additional time, this cost is incurred only once. After the MLP model is established, its prediction time is negligible compared with that of the FEM when the nonlinear B–H characteristic is considered. Therefore, the MLP model can effectively replace the FEM for repeated evaluations in the superellipse-curve-based topology optimization process.

**Figure 9:**
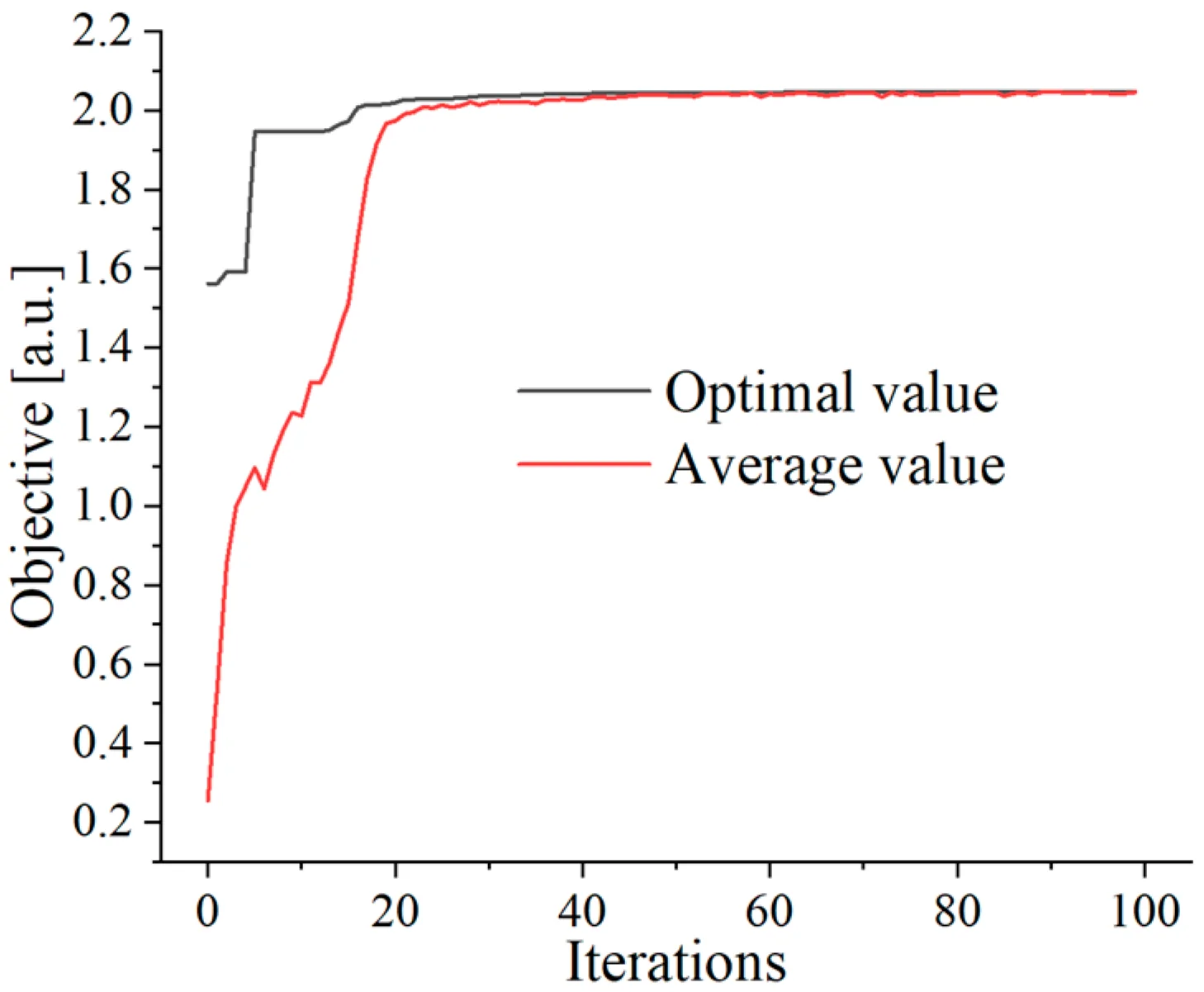
Convergence curves of genetic algorithm.

When the reference magnetic force *F_x_ref_* is set to 53.83 N/m, the convergence histories of the genetic algorithm, including the optimal objective value and the average objective value in each generation, are shown in Figure 9. It can be observed that the objective value gradually converges as the number of generations increases, and the optimal value becomes relatively stable after approximately 30 generations. The optimized maximum relative objective function is about 2.040.

**Figure 10:**
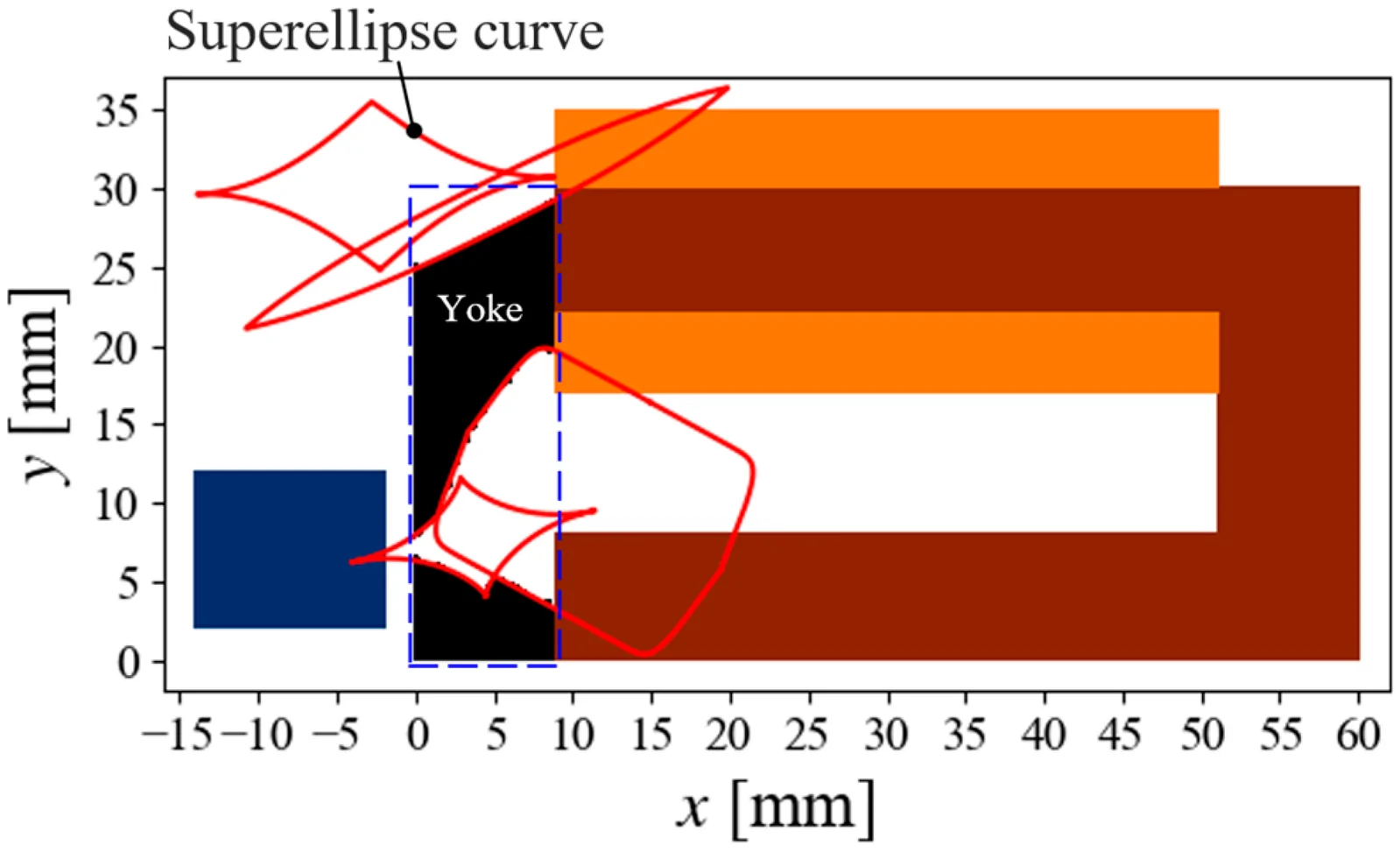
Optimal yoke distribution based on topology optimization.

Based on the optimization results shown in Figure 9, the optimal yoke topology within the design domain is presented in Figure 10, where the black region represents the yoke and the white region denotes air. Overall, the final optimized yoke exhibits a C-shaped configuration. However, some local differences can be observed compared with the topology obtained under the linear-yoke assumption, as shown in Figure 6, owing to the influence of the nonlinear B–H characteristic. Moreover, because one superellipse curve does not appear in the final optimized boundary, four superellipse curves are sufficient to construct the topology optimization framework for the magnetic actuator.

For the fixed-mesh yoke topology shown in Figure 10, the FEM-calculated magnetic force and yoke area are approximately 56.38 N/m and 151.91 mm^2^, respectively. According to Equation (12), the corresponding relative objective function is approximately 2.047. Therefore, the relative difference between the predicted and calculated values for the optimized yoke topology is about 0.34%, indicating good agreement.

To further improve the reliability of the optimization results, the surrogate model can be used for preliminary or coarse optimization to rapidly narrow the design space and identify promising candidate regions. Subsequently, a high-fidelity FEM model can be employed for local refinement and final optimization to obtain a more reliable optimum.

**Figure 11:**
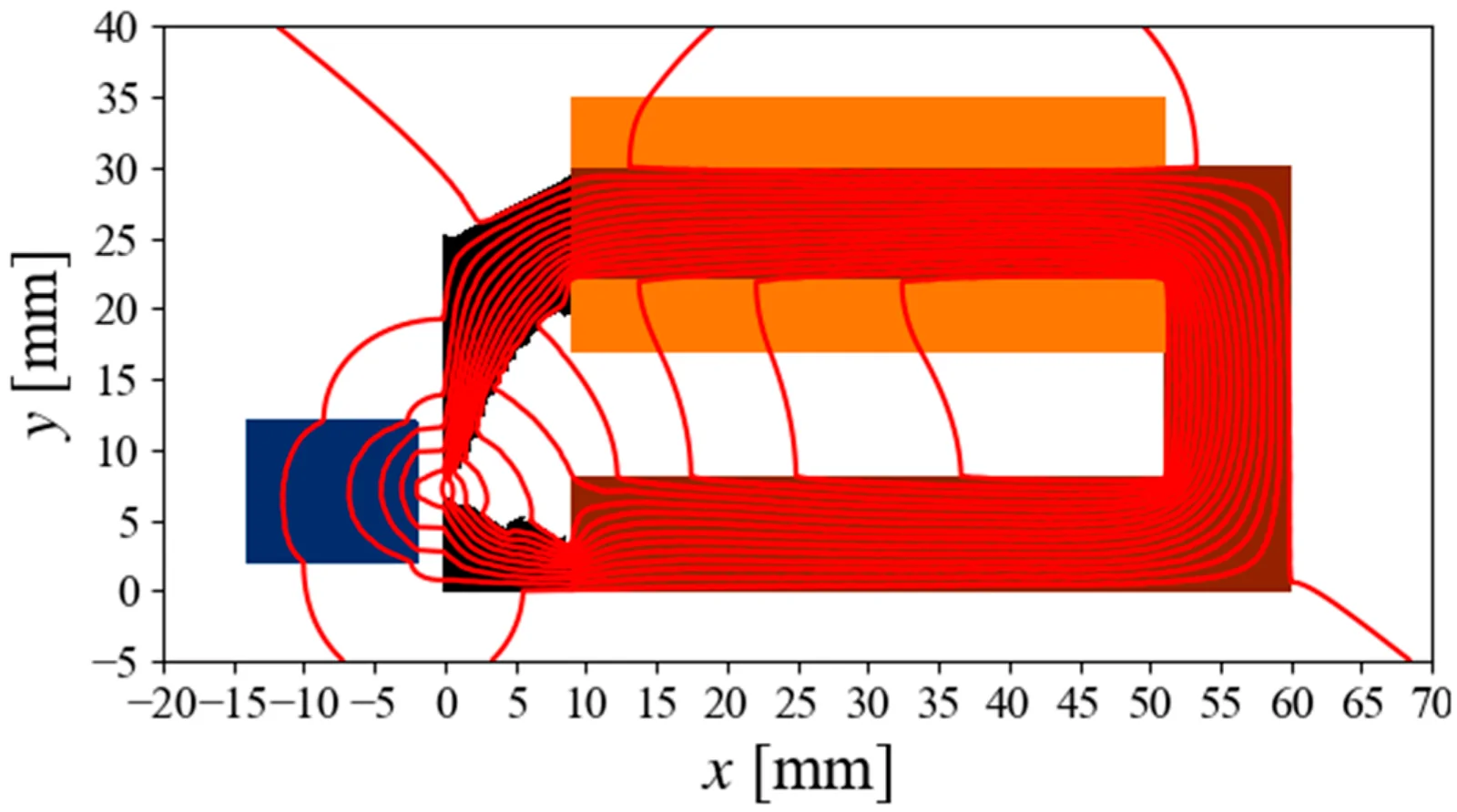
Magnetic flux path distribution of the optimized magnetic actuator.

The distribution of the magnetic flux paths is shown in Figure 11. It can be seen that the optimized yoke effectively guides the magnetic field generated by the coil toward the armature, thereby enhancing the magnetic force.

Now, the fabrication convenience of the optimized yoke topology should be considered. In Figure 10, the topology is represented using a fixed mesh, which may result in irregular and non-smooth boundaries. In fact, because the material boundaries are directly described by superellipse curves and each superellipse curve has an explicit analytical expression, the optimized yoke topology can be conveniently extracted without additional boundary fitting or approximation.

**Figure 12:**
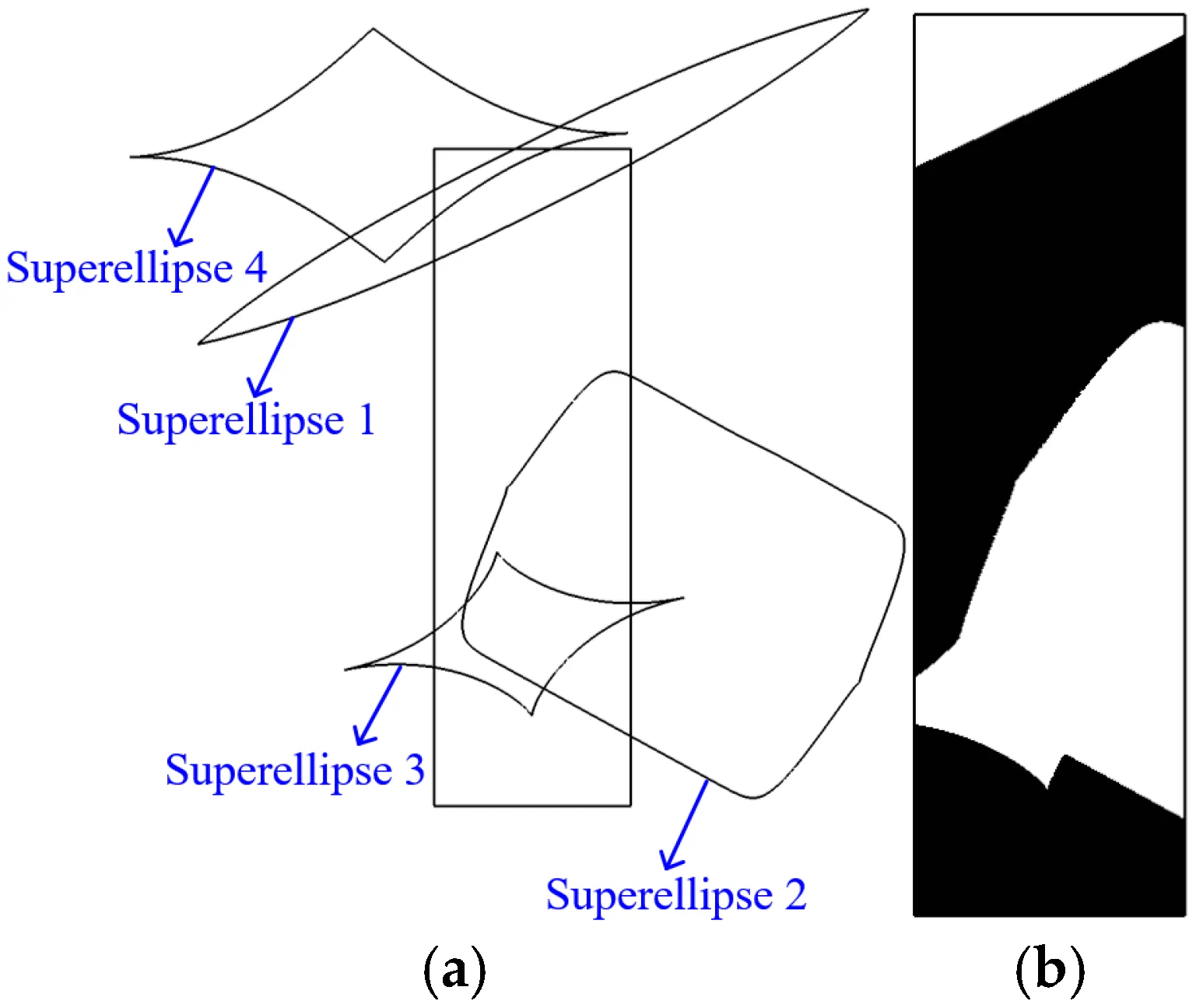
Yoke topology directly based on the four superellipse curves. (**a**) Relative positions of superellipses and design domain boundary. (**b**) Yoke topology based on the superellipses.

Figure 12a shows the relative positions of the four optimized superellipse curves and the design-domain boundaries. Based on these superellipse curves, the final yoke topology boundaries can be directly extracted, as shown in Figure 12b, resulting in clearer and smoother boundaries. It can be seen that the final optimized yoke has well-defined, smooth boundaries, which is beneficial for manufacturing.

For the yoke topology defined by the superellipse curves, the calculated magnetic force and yoke area are approximately 56.27 N/m and 151.80 mm^2^, respectively. Notably, the relative difference in magnetic force between the fixed-mesh-based and superellipse-based yoke topologies is only about 0.20%. Therefore, the superellipse-based yoke topology, with its smooth and well-defined boundaries, can be directly extracted for final fabrication.

To compare the topologies optimized under the linear- and nonlinear-yoke assumptions, Table 2 summarizes the corresponding magnetic force and yoke area. In both cases, the topologies were obtained using a topology optimization framework based on four superellipse curves. For the linear-yoke case, the resulting magnetic force and area fraction are 53.83 N/m and 0.56, respectively.

For the yoke topology optimized with the nonlinear material characteristic considered, the corresponding magnetic force and area fraction, as listed in Table 2, are 56.38 N/m and 0.56, respectively. The magnetic force is approximately 4.74% higher than that obtained under the linear-yoke assumption. Therefore, to enhance the magnetic force of the armature in the magnetic actuator, the nonlinear B–H characteristic of the yoke should be taken into account.

Next, we need to explore the numerical uncertainties of the performance difference between the optimized linear yoke and nonlinear yoke, and the initial fixed mesh should be changed to a finer mesh and more nonlinear iterations should be adopted. Specifically, the optimized yoke boundaries are more accurately described by the superellipse curves for both the linear yoke and the nonlinear yoke. For the optimized linear yoke, the meshed elements in the design domain increased from 1864 to 4352. For the optimized nonlinear yoke, the meshed elements in the design domain increased from 1864 to 4375, and the threshold for the nonlinear iteration was reduced to ensure more accurate calculation results. As a result, the calculated force for the linear yoke changed from 53.83 N/m to 53.75 N/m, and the relative difference is about 0.15%. Furthermore, the calculated force for the nonlinear yoke changed from 56.38 N/m to 56.27 N/m, and the relative difference is about 0.20%. The results calculated using the initial fixed mesh exhibit accuracy comparable to that obtained using a finer mesh. This can be attributed to the use of second-order triangular elements in the FEM analysis and the relatively fine resolution of the initial mesh. Moreover, based on the initial fixed mesh, the improvement in force for the optimized nonlinear yoke is about 4.74% compared with that for the optimized linear yoke. However, when the yoke boundaries are directly represented by superellipse curves with a finer mesh and more accurate nonlinear iteration, the improvement is about 4.48%, and the comparison results are relatively stable.

It should be noted that the reported improvement in the magnetic force of the actuator is a numerical prediction based on the assumptions of the FEM model. Discrepancies between the numerical results and the performance of the physical actuator may arise from uncertainties in the magnetic material properties, as well as manufacturing and assembly tolerances. These factors are beyond the scope of the present study.

Based on the above analysis, it can be concluded that the proposed superellipse-curve-based gradient-free topology optimization method can be efficiently applied to the design of electromagnetic devices. It is applicable to problems involving both linear and nonlinear materials, as well as complex objective functions. Moreover, with the aid of the adopted MLP-based surrogate model, the time-consuming nonlinear B–H calculations based on FEM can be avoided, reducing the evaluation time for a single magnetic actuator topology from about 16.43 s to a negligible level. As a result, the genetic-algorithm-based topology optimization can be completed efficiently within several minutes.

## 6. Conclusions

In this work, an efficient gradient-free topology optimization method that integrates superellipse curves with a multilayer perceptron (MLP) surrogate model is proposed. By exploiting the strong deformation capability of superellipse curves with only a limited number of control parameters, the proposed method enables topology optimization without requiring complex sensitivity derivations and is well suited to problems with complex objective functions and nonlinear materials. The lightweight MLP-based surrogate model achieves a Pearson correlation coefficient of 0.99 relative to the FEM results, reducing the time required for nonlinear B–H calculations of the magnetic actuator from approximately 16.43 s to a negligible level. Consequently, the total topology optimization time is reduced by approximately 82.8% compared with direct FEM-based genetic algorithm optimization. The topology optimization results further demonstrate that the proposed method is applicable to both linear and nonlinear materials. Under the nonlinear-yoke condition, the optimized magnetic force is approximately 4.74% higher than that under the linear-yoke condition, highlighting the practical importance of considering nonlinear material characteristics in the topology optimization of magnetic actuators.

Nevertheless, the proposed framework still has certain limitations. Although the superellipse-based parameterization provides a compact and flexible representation of material boundaries, representing highly irregular or multiscale topologies, or topologies with slender branches, may require an increased number of superellipse curves. This would enlarge the design space and consequently increase the costs of sample generation, surrogate model training, and optimization. Future work will therefore focus on developing more adaptive and hierarchical geometric representations, together with efficient sampling and surrogate model updating strategies, to improve the capability to represent complex topologies while maintaining computational efficiency and to extend the proposed method to a broader range of operating conditions and engineering scenarios.

Finally, because experimental measurements of a manufactured prototype of the optimized actuator are not available under the current experimental conditions, the experimental fabrication and measurement of the optimized device, as well as the consideration of material-property uncertainties, manufacturing tolerances, and other implementation-related factors, are beyond the scope of the present study. These aspects will be addressed in future work.

## Figures and Tables

**Table 1:** Ranges of parameters for each superellipse curve.

Parameters	Ranges
*a*	(0, 9]/[mm]
*b*	(0, 30]/[mm]
*p*	[0.1, 4]
*q*	[0.1, 4]
*α*	[0, 2π]
*x*_0_	[−9, 18]/[mm]
*y*_0_	[−30, 60]/[mm]

**Table 2:** Performance of yoke optimized under different cases.

Optimization Case	Magnetic Force [N/m]	Area Fraction
Linear yoke	53.83	0.56
Nonlinear yoke	56.38	0.56

## Data Availability

The data that support the findings of this manuscript are available from the corresponding author upon reasonable request.
